# Systemische Auswirkungen und klinische Aspekte der SARS-CoV-2-Infektion

**DOI:** 10.1007/s00292-021-00913-0

**Published:** 2021-02-11

**Authors:** Sigurd F. Lax, Kristijan Skok, Peter M. Zechner, Lisa Setaffy, Harald H. Kessler, Norbert Kaufmann, Klaus Vander, Natalija Cokić, Urša Maierhofer, Ute Bargfrieder, Michael Trauner

**Affiliations:** 1 Institut für Pathologie des LKH Graz II, Akademisches Lehrkrankenhaus der Medizinischen Universität Graz, Göstingerstrasse 22, 8020 Graz, Österreich; 2 Medizinische Fakultät, Johannes-Kepler-Universität Linz, Linz, Österreich; 3 Abteilungen für Innere Medizin, LKH Graz II, Graz, Österreich; 4 Diagnostik und Forschungsinstitut für Hygiene, Mikrobiologie und Umweltmedizin, Medizinische Universität Graz, Graz, Österreich; 5 Institut für Krankenhaushygiene und Mikrobiologie, Steiermärkische Krankenanstaltengesellschaft m.b.H., Graz, Österreich; 6 Abteilungen für Anästhesiologie und Intensivmedizin, LKH Graz II, Graz, Österreich; 7 Klinische Abteilung für Gastroenterologie und Hepatologie, Intensivstation 13H1, Universitätsklinik für Innere Medizin III, Medizinische Universität/AKH Wien, Wien, Österreich

**Keywords:** Autopsie, COVID-19, Ischämische Darmschädigung, Lungenversagen, Pulmonale Thrombose, Autopsy, COVID-19, Ischemic bowel disease, Respiratory distress syndrome, Pulmonary thrombosis

## Abstract

**Hintergrund:**

COVID-19 wird als systemische Erkrankung eingestuft. Ein schwerer Verlauf mit tödlichem Ausgang ist möglich und unvorhersehbar.

**Fragestellung:**

Welche Organsysteme sind primär betroffen? Welche Organveränderungen prädisponieren für einen ungünstigen Verlauf? Welche Organschädigungen finden sich bei letalem Ausgang?

**Material und Methode:**

Daten aus publizierten Obduktionsstudien (davon 28 eigene publizierte Fälle) in Hinblick auf Organschädigung und mögliche Todesursachen.

**Ergebnisse:**

Die schwersten Veränderungen finden sich in den Lungen in Form eines diffusen Alveolarschadens als akutes Atemnotsyndrom des Erwachsenen (ARDS), zum Teil bereits mit Fibrose. Thrombosen in kleinen bis mittelgroßen Pulmonalarterien sind mit Lungeninfarkten vergesellschaftet. Häufige Komplikationen sind bakterielle Bronchopneumonien, seltener Pilzpneumonien. Pulmonale Thromboembolien finden sich in 20–30 % der tödlichen Verläufe, auch bei Fehlen einer tiefen Beinvenenthrombose. Eine intestinale Beteiligung von COVID-19 kann mit ischämischer Schädigung des Darmes einhergehen, in erster Linie bedingt durch Schock oder lokale Thrombose. Die Nieren zeigen eine akute Tubulusschädigung als Ausdruck eines akuten Nierenversagens, Lymphknoten und Milz einen Schwund der Lymphozyten, die Nebennierenrinde eine Hyperplasie. In der Leber finden sich häufig eine Steatose, Leberzellnekrosen, ein portales Entzündungsinfiltrat und eine Proliferation der Kupffer-Zellen. Häufige Grunderkrankungen sind in den Autopsiekollektiven arterieller Hypertonus mit hypertensiver und ischämischer Kardiomyopathie und Diabetes mellitus. In großen bevölkerungsbasierten Studien ergibt sich aber für Hypertoniker im Gegensatz zu Diabetikern kein erhöhtes Mortalitätsrisiko.

**Schlussfolgerungen:**

Pulmonale Kreislaufstörungen mit arteriellen Thrombosen, Infarkten und Pneumonien sind wesentliche und oft letale Komplikationen des ARDS bei COVID-19. Die Erkenntnisse aus Obduktionsstudien haben Therapie und Prophylaxe beeinflusst.

## Abstract (en)

**Background:**

COVID-19 is considered a systemic disease. A severe course with fatal outcome is possible and unpredictable.

**Objectives:**

Which organ systems are predominantly involved? Which diseases are predisposed for a fatal course? Which organ changes are found with lethal outcome?

**Materials and methods:**

Data from published autopsy studies (28 cases by our group) with respect to organ changes and possible cause of death.

**Results:**

The most severe alterations are found in the lungs by diffuse alveolar damage as a symptom of an acute respiratory distress syndrome (ARDS), in part with fibrosis. Thrombosis of small- to mid-sized pulmonary arteries is associated with hemorrhagic lung infarction. Frequent complications are bacterial pneumonias and less frequently fungal pneumonias by aspergillus. Pulmonary thromboembolism is found in 20–30% of lethal courses, also in the absence of deep venous thrombosis. Intestinal involvement of COVID-19 can be associated with intestinal ischemia, caused by shock or local thrombosis. In most cases, the kidneys display acute tubular injury reflecting acute renal failure, depletion of lymphocytes in the lymph nodes and spleen, and hyperplastic adrenal glands. The liver frequently reveals steatosis, liver cell necrosis, portal inflammation, and proliferation of Kupffer cells. Important preexisting diseases in autopsy studies are arterial hypertension with hypertensive and ischemic cardiomyopathy and diabetes mellitus but large population-based studies reveal increased risk of mortality only for diabetes mellitus not for arterial hypertension.

**Conclusions:**

Alterations of the pulmonary circulation with pulmonary arterial thrombosis, infarction, and bacterial pneumonia are important and often lethal complications of COVID-19-associated ARDS. Findings from autopsy studies have influenced therapy and prophylaxis.

Die durch das neue Coronavirus SARS-CoV‑2 verursachte Erkrankung COVID-19 ist durch unterschiedliche Krankheitsverläufe und Symptome charakterisiert [13]. Am häufigsten ist ein asymptomatischer oder milder Verlauf. Typischerweise ist COVID-19 mit grippeartigen Allgemeinsymptomen vergesellschaftet, kann aber auch gastroenteritis- oder hepatitisartige Manifestationen zeigen.

Bei schwerem oder kritischem Verlauf bietet sich ein Sepsis/SIRS(„systemic inflammatory response syndrome“)-artiges Bild mit schwerer Schädigung der Lungen und weiteren Organmanifestationen. Eine ausschließlich extrapulmonale Symptomatik ist hierbei ungewöhnlich. Der folgende Übersichtsartikel soll die verschiedenen Organveränderungen in Bezug zum klinischen Bild beleuchten, wobei die Daten vor allem auf von uns und anderen Gruppen publizierten Obduktionsstudien basieren (Tab. 1).

**Table 1:** 

Erstautoren	Ort (Land)	Anzahl Fälle	Publiziert (Monat/Jahr)	Literaturzitate
Lax, Skok	Graz, LKH II (AT)	11/19/28	5/2020, 8 und 12/2020	[19, 39, 40]
Menter	Basel, Liestal (CH)	21	5/2020	[24]
Wichmann, Edler	Hamburg (D)	12/80^a^	5/2020, 6/2020	[23, 48]
Schaller	Augsburg (D)	10	5/2020	[36]
Bösmüller	Tübingen (D)	4	6/2020	[3]
Bradley	Seattle, Everett (USA)	14	7/2020	[4]
Hanley	London (UK)	10	8/2020	[11]

^a^nur 12 Fälle mit histologischer Untersuchung

## Primär betroffene Organe: Lungen und Gastrointestinaltrakt

Eine Infektion mit SARS-CoV‑2 betrifft primär die Lungen und andere Teile des Respirationstraktes sowie häufig den Gastrointestinaltrakt (GI-Trakt). Dementsprechend liegt häufig eine grippeartige Symptomatik mit Müdigkeit, Fieber und respiratorischen Symptomen, vor allem trockenem Husten vor. Ein Teil der Patienten (ca. 10–20 %) zeigt aber primär eine gastrointestinale Symptomatik mit enteritisartigem Bild mit Durchfällen, aber auch ein hepatitisartiges Krankheitsbild wurde beschrieben [41]. Interessanterweise wiesen bis zu 28 % der Patienten mit gastrointestinalen Symptomen keine respiratorischen Symptome auf [15]. Das Ausmaß der Organschädigung unterscheidet sich deutlich zwischen Lungen und Darm (Tab. 2).

**Table 2:** 

Organ	Veränderung	Häufigkeit (%)	Literaturzitate
Lungen	Diffuser Alveolarschaden Arterielle (Mikro‑)Thrombosen Bronchopneumonie Pilzpneumonie Pulmonale Thrombembolien Hämorrhagischer Infarkt	66–100 29–100 33–55 <5 14–33 17–82	[3, 4, 19, 24, 36, 40, 48] [3, 4, 11, 19, 24, 48] [11, 19, 24, 40, 48] [40] [4, 23, 24, 40, 48] [19, 24, 48]
Darm	Ischämische Schädigung	24	[40]
Nieren	Akute Tubulusschädigung	93–100	[19, 24]
Herz	Herzmuskelnekrosen Myokarditis	9–14 0–9	[19, 24] [4, 19, 36, 48]
Leber	Steatose Leberzellnekrosen Portale Entzündung Thrombose	59–100 64 29–73 10	[4, 19, 24] [19] [4, 19] [19]
Pankreas	Akute Pankreatitis (fokal)	14–45	[11, 19, 40]
Lymphknoten	Verminderung der Lymphozyten Vermehrung von Plasmablasten	100 56	[11, 19] [24]
Milz	Atrophie der weißen Pulpa	91–100	[11, 19]
Nebenniere	Diffuse und/oder nodöse Hyperplasie der Rinde	75	[19]
Venen	Tiefe Bein‑/Beckenvenenthrombosen	10–58	[40, 48]

**Figure 1:**
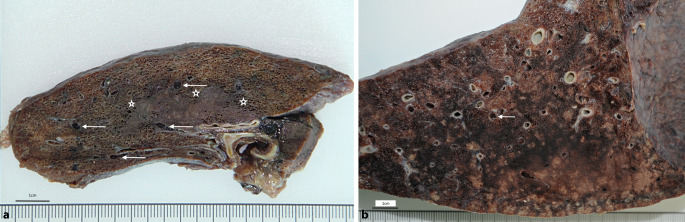

**Figure 2:**
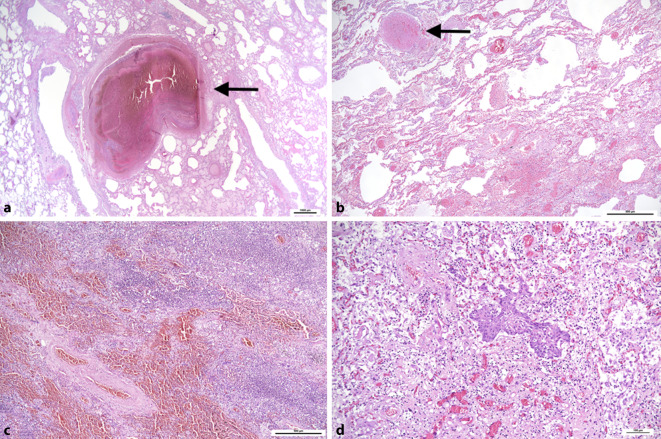

Die Lungen weisen bei schwerem Verlauf das Bild eines akuten Atemnotsyndroms (acute respiratory distress syndrom/ARDS) mit einem diffusen Alveolarschaden auf. Dieser ist durch unterschiedliche Stadien charakterisiert, die nebeneinander bestehen können, wodurch makroskopisch und histologisch meist ein inhomogenes Bild entsteht. Im Zuge der frühen exsudativen Phase kommt es vorerst zu einer massiven Stauung im Bereich der alveolären Kapillaren, einem Ödem und zur Ausbildung hyaliner Membranen im Bereich der Wand der Alveolen, in der Folge zu einer Proliferation von Makrophagen, die das Lumen der Alveolen ausfüllen können [19, 24]. Die Resorption dieses entzündlichen Infiltrates führt meist zu einer Fibrose unter dem Bild der organisierenden Pneumonie. Dabei können auch Nester metaplastischen Plattenepithels im Bereich der ehemaligen Alveolen auftreten (Abb. 2d) [3]. Ein wesentlicher und für die Prognose entscheidender Befund sind allerdings thrombotische Gefäßverschlüsse auf der Ebene subsegmentaler und segmentaler Pulmonalarterienäste (Abb. 1 und 2a, b; [3, 19]). Diese sind typisch für das ARDS bei COVID-19 und möglicherweise durch eine Endotheliitis mitverursacht [2, 43]. Da auch sehr kleine Gefäße betroffen sein können und die Veränderungen ursprünglich mikroskopisch entdeckt wurden, wird in der Literatur fälschlicherweise von Mikrothromben gesprochen, obwohl ein Teil dieser Thromben makroskopisch erkennbar ist [30]. Ebenso wird die Genese der pulmonalen Blutgerinnsel oft nicht exakt dargestellt, indem lokale Thrombosen auf subsegmentaler Ebene mit Thromboembolien verwechselt bzw. als solche fehlinterpretiert werden. Es finden sich auch Blutungen in das Lungengewebe, die Ausdruck lokaler Durchblutungsstörungen sein können. Die Häufigkeit eines diffusen Alveolarschadens verbunden mit Thrombosen scheint den Veränderungen bei SARS‑1 zu ähneln [8, 12]. In unserem Untersuchungsgut fanden sich in ca. 20 % der Fälle auch Thromboembolien in größeren Pulmonalarterienästen, wobei nur etwa in der Hälfe der Fälle eine tiefe Beinvenenthrombose nachweisbar war. In der ersten Hamburger Studie waren pulmonale Thromboembolien mit etwa 33 % etwas häufiger, noch häufiger fanden sich hingegen tiefe Beinvenenthrombosen (knapp 60 %) [48]. In einer Folgestudie zeigten sich pulmonale Thromboembolien in 21 % und tiefe Beinvenenthrombosen in 40 % [23]. Pulmonalarterielle Thrombosen und Thromboembolien führten in unserem Untersuchungsgut häufig zu hämorrhagischen Lungeninfarkten unterschiedlicher Ausdehnung, oft nur mit wenigen Zentimetern Durchmesser. Infarkte, Lungenparenchymblutungen und die massive Hypostase mit oft infarktartigen Zirkulationsstörungen, letztere in Verbindung mit einer linksventrikulären Insuffizienz begünstigen die Entstehung bakterieller Bronchopneumonien mit betont neutrophil-granulozytärem Exsudat (Abb. 2c). Pilzpneumonien, speziell durch *Aspergillus* wurden bei bis zu einem Drittel der intensivpflichtigen COVID-19-Patienten beschrieben und als COVID-assoziierte pulmonale Aspergillose (CAPA) bezeichnet, fanden sich hingegen nur selten in unserem Obduktionskollektiv (in < 5 %) [44]. Die beschriebenen Lungenveränderungen führen je nach vorhandener Reservekapazität früher oder später zu massiven Problemen bei der Oxygenierung und sind somit auch für die respiratorischen Probleme verantwortlich. Bronchopneumonien, Lungeninfarkte sowie Thrombosen und Thromboembolien können als wesentliche Todesursachen betrachtet werden [23, 40]. Vorbestehende Erkrankungen bzw. Gewebeschädigungen sind im Einzelfall als Todesursache oder wesentliche Kofaktoren zu berücksichtigen [5]. Nach durchgemachter SARS-CoV-2-Infektion sprechen die schweren Lungenveränderungen, wie z. B. ein ARDS mit Lungenarterienthrombosen, auch ohne unmittelbaren postmortalen Nachweis viraler RNA für eine COVID-19-assoziierte und für COVID-19 typische Lungenveränderung [39].

**Figure 3:**
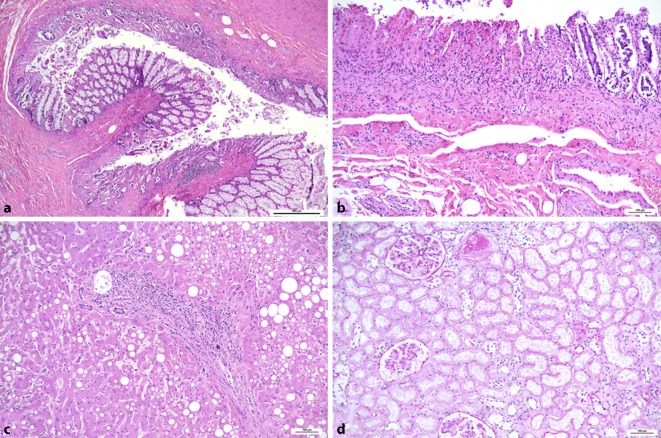

Im Gegensatz dazu ist die morphologische Basis der gastrointestinalen Symptome, welche in ca. 10–20 % der Patienten mit COVID-19 vorliegen, schlecht charakterisiert [29]. Der Nachweis von SARS-CoV‑2 in der intestinalen Mukosa und im Stuhl legt nahe, dass der GI-Trakt eine mögliche Infektionsroute darstellen könnte [15]. Wir fanden bei einem Teil der an COVID-19 Verstorbenen ischämische Darmschädigungen vergesellschaftet mit einem Nachweis viraler RNA in der Darmschleimhaut [39]. Die ischämischen Veränderungen sind überwiegend durch flache Schleimhautulcera charakterisiert (Abb. 3a, b) und in erster Linie im Rahmen des Schockgeschehens und weniger durch eine lokale Koagulopathie mit Mikrothromben zu erklären. Ungeklärt ist, ob SARS-CoV‑2 die Darmschleimhaut durch Vermehrung in den Enterozyten direkt schädigt. Bis dato gibt es auch keine Berichte über eine schwere und nachhaltige Schädigung des GI-Traktes mit Defektheilung. Auch bezüglich akuter schwerer bzw. letaler Verläufe durch Befall des Gastrointestinaltraktes gibt es eingeschränkte Evidenz. In letzter Zeit wurden akute ischämische Komplikationen von COVID-19-Patienten berichtet, unter anderem durch mesenterielle Gefäßthrombosen, die eine akute Laparotomie erforderlich machten [38]. Ein erhöhter Bedarf an Opioiden und die durch COVID-19 induzierte Koagulopathie könnten auch die unverhältnismäßig hohe Rate an Ileus und ischämischer Darmschädigung erklären [6].

## Weitere Organveränderungen aufgrund viraler Sepsis und Schockgeschehen

Im Zuge einer schweren COVID-19-Erkrankung kann eine Reihe weiterer Organe betroffen sein (Tab. 2), sodass sich der Begriff einer systemischen Erkrankung durchaus anbietet [20]. Dies wird auch immer wieder von erkrankten Personen nach deren Genesung zum Ausdruck gebracht (persönliche Mitteilungen). Eine Reihe von Organveränderungen sind wahrscheinlich auf den Kreislaufschock im Rahmen der viralen Sepsis zurückzuführen, der die systemische Wirkung des „Zytokinsturms“ noch verstärkt. Da sich Gewebeuntersuchungen bis auf Ausnahmen auf Autopsiematerial beschränken, besteht unser Wissen fast ausschließlich über Veränderungen bei letalem Verlauf. Bei leichteren und mittelschweren Krankheitsverläufen gibt es zwar reichlich klinische Informationen, aber kaum Daten zu eventuellen morphologischen Organveränderungen [10].

In nahezu allen Fällen mit letalem Ausgang zeigt sich eine akute Schädigung der Nierentubuli („acute tubular injury“), die in erster Linie als schockbedingt zu werten ist (Abb. 3d). Akute Veränderungen des Myokards wie Herzmuskelnekrosen finden sich nur in wenigen Fällen [19], ebenso ein entzündliches Infiltrat im Sinne von Myokarditiden [4] obwohl Virus-RNA in etwa 60 % der Fälle im Herzmuskel nachweisbar ist (davon in 20 % in sehr geringen Mengen) [21]. Dies steht im Gegensatz zu klinischen Berichten über eine häufige Beteiligung des Herzens bei COVID-19 [31]. Leberzellnekrosen sind hingegen nicht ungewöhnlich und können ebenfalls als schockbedingt bzw. im Rahmen einer hypoxischen Hepatitis (bei ARDS) betrachtet werden. In der Leber fanden sich häufig eine Steatose (Abb. 3c), Leberzellnekrosen, ein portales Entzündungsinfiltrat und eine Proliferation der Kupffer-Zellen [19], wobei diese Veränderungen Ausdruck einer direkten zytopathischen Schädigung durch Infektion mit SARS-CoV‑2, des Zytokinsturms, der Hypoxie durch ARDS bzw. vaskuläre Veränderungen, aber auch iatrogener Ursachen (Medikamente, hoher Beatmungsdruck) sein können [26]. Eine herdförmige akute Pankreatitis, ebenfalls in erster Linie schockbedingt, fand sich in unserer ersten Obduktionsserie in 45 %, in einer Obduktionsserie aus England in 25 % der Fälle, ebenso in klinischen Berichten (in bis zu 12 % der Patienten) [9]. In der Erweiterung unserer Obduktionsserie fanden sich aber kaum Pankreatitiden [39, 40], ebenso wenig wurde von anderen Gruppen über pathologische Veränderungen des Pankreas berichtet [24, 48].

## Veränderungen des lymphatischen und des endokrinen Systems

**Figure 4:**
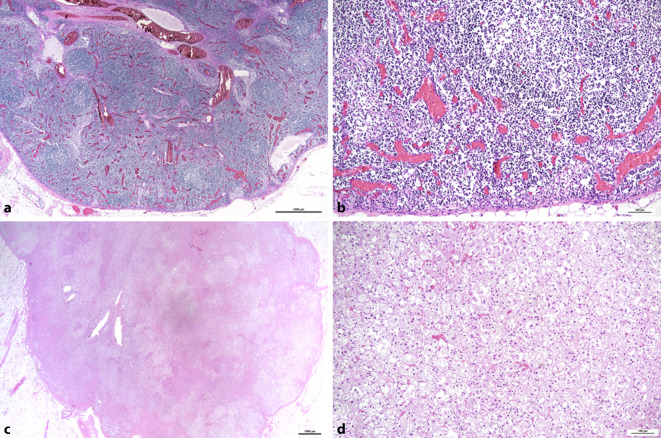

COVID-19 ist in seinen schweren Verläufen mit einer ausgeprägten Lymphopenie vergesellschaftet, die sich auf organischer Ebene als Verminderung der weißen Pulpa der Milz und der Lymphozyten in den Lymphknoten äußert [19]. Dabei erscheinen die Lymphknoten zwar makroskopisch als groß, bei der histologischen Untersuchung ist das lymphatische Gewebe aber stark reduziert und Keimzentren fehlen (Abb. 4a, b). Kompensatorisch sind die Sinus deutlich ausgeweitet, es können auch Makrophagen vermehrt sein [11, 19]. Die „Erschöpfung“ der Lymphozyten betrifft vor allem CD8-positive T‑Lymphozyten [11]. Andererseits können auch Plasmablasten vermehrt sein [24].

Das endokrine System zeigt häufig eine diffuse und mitunter nodöse Hyperplasie der Nebennierenrinde (Abb. 4c, d). Der dadurch bedingte Hypercortisolismus wird für die Reduktion der Lymphozyten verantwortlich gemacht [28]. Dazu gibt es ähnliche Berichte von SARS-CoV‑1 [47]. Für die Hyperplasie der Nebennierenrinde wird ein durch die systemische Erkrankung bedingter hypothalamisch-hypophysär gesteuerter Stressmechanismus verantwortlich gemacht, der genaue Pathomechanismus ist aber nicht bekannt.

## Morphologische und funktionelle Veränderungen des Nervensystems

Im Zusammenhang mit COVID-19 wurden verschiedene neurologische Störungen berichtet, die mit unterschiedlichen Symptomen einhergehen. Als frühe Symptome werden Kopfschmerzen, Schwindelgefühl sowie Störungen des Geschmacks‑ und des Geruchsempfindens eingestuft [33]. Auch für die Entstehung der Ateminsuffizienz wird eine neurogene Komponente und Genese vermutet. Im Wesentlichen wird angenommen, dass sich das Virus über den Nervus olfactorius in den Hirnstamm und das dort gelegene Atemzentrum ausbreitet [33]. Eine COVID-assoziierte Enzephalitis ist selten, kann aber letal verlaufen [42]. Virale RNA kann dabei im Liquor entdeckt und bei Obduktionen kann zusätzlich auch Virusprotein im Gehirngewebe nachgewiesen werden [23, 42]. Zerebrovaskuläre Veränderungen, die mit arteriellen und venösen Thrombosen, Infarkten und Blutungen einhergehen, sind durch die COVID-assoziierte Koagulopathie und Endotheliitis wesentlich mitverursacht [7]. Nicht zuletzt ist auf mögliche Nebenwirkungen der medikamentösen Therapie von COVID-19 auf das Nervensystem zu achten. Es gibt ferner Hinweise darauf, dass gerontopsychiatrische Patienten häufiger an COVID-19 erkranken mit erhöhtem Risiko für einen schwereren Verlauf [27].

## Organveränderungen als mögliche Prädisposition für COVID-19

Aufgrund des hohen Altersschnittes (80 Jahre) der Patienten mit letalem Verlauf fanden sich in den Autopsiekollektiven häufig Erkrankungen aus dem Bereich der chronischen degenerativen Gefäßerkrankungen wie allgemeine Arteriosklerose, stenosierende Koronararteriensklerose mit ischämischer Kardiomyopathie, benigne Nephrosklerose und linksventrikuläre Myokardhypertrophie im Sinne einer hypertensiven Kardiomyopathie. Das tatsächliche Mortalitätsrisiko dieser Erkrankungen in Bezug auf COVID-19 scheint aber anders gelegen zu sein. In einer großen Studie an mehr als 10.000 COVID-assoziierten Todesfällen ergab sich ein erhöhtes Mortalitätsrisiko für Diabetes mellitus, Alter über 60 Jahre, rezente Tumorerkrankungen und Zustand nach Organtransplantation, aber nicht für arteriellen Hypertonus [49]. Eine isolierte kardiale Amyloidose zeigte sich in 14–29 %, wobei ein ursächlicher Zusammenhang mit COVID-19 zwar ungeklärt, aber eher unwahrscheinlich ist [4, 24, 40]. Bei einem Teil der Patienten (30–40 %) fand sich außerdem ein zum Teil insulinpflichtiger Diabetes mellitus Typ II. Es ist möglich, dass eine mit ischämischer bzw. hypertensiver Kardiomyopathie einhergehende chronische Herzinsuffizienz die Entstehung der pulmonalarteriellen Zirkulationsstörung mit Ausbildung von Thromben begünstigt. Eine arterielle Hypertonie findet sich bei etwa 80 % der an COVID-19 Verstorbenen. Inwieweit die Dichte an ACE-Rezeptoren bzw. eine mögliche antihypertensive Therapie hierbei eine Rolle spielt, ist ungeklärt. Nach neueren Daten stellt eine Therapie mit Inhibitoren des Renin-Angiotensin-Aldosteron-Systems jedoch keinen Risikofaktor dar [1, 22].

## Dauerhafte Organschäden als Folge von COVID-19

Über die Dauerfolgen bei überstandener COVID-19 Erkrankung (Post-COVID-Syndrom) gibt es zunehmend mehr Daten und Erkenntnisse. In etwa 70 % der Patienten mit pulmonaler Manifestation von COVID-19 mit diffusem Alveolarschaden finden sich Lungenfibrosen als Ausdruck einer Defektheilung [14, 50]. Dabei ist die Diffusionskapazität der Lungen in der Regel um 50 % reduziert. Häufig finden sich auch Herzmuskelveränderungen im MRT und manifeste Myokarditiden, unter anderem bei erkrankten aktiven Sportlern [31, 32]. Über Dauerschäden des Herzens gibt es hingegen keine Erfahrungswerte. Psychische Veränderungen finden sich in Form von chronischer Müdigkeit und Hoffnungslosigkeit, aber auch Isolation und Einsamkeit und werden als „brain fog“ bezeichnet [51]. Schlussendlich scheint es auch nach mildem Krankheitsverlauf unterschiedliche immunologische Reaktionsformen zu geben, wobei die einen günstigerweise mit einer Aktivierung von T‑Zellen und erhöhter Anzahl von Plasmablasten, die anderen ungünstigerweise mit einer Reduktion der neutrophilen Granulozyten und der regulatorischen T‑Lymphozyten einhergehen [17]. Informationen über einen möglichen Zusammenhang zwischen immunologischem Profil und akuten sowie dauerhaften Organschäden fehlen jedoch noch.

## Klinische Konsequenzen aus den Obduktionsstudien

Die Ergebnisse der Obduktionsstudien haben wesentlichen Einfluss auf das therapeutische Management der COVID-19-Patienten [18, 35]. Eine Thromboseprophylaxe wird bei allen symptomatischen Patienten ab dem Erkrankungsbeginn empfohlen und hat möglicherweise zu einer Verminderung der Häufigkeit letaler Verläufe beigetragen [25]. Ein weiterer wesentlicher Punkt ist eine gezielte Antibiose zur Vermeidung bzw. Therapie bakterieller Pneumonien, die eine wesentliche Komplikation und häufige Todesursache darstellen. Bei Intensivpatienten wird das Screening auf Pilzinfektionen mit entsprechendem Einsatz einer antimykotischen Therapie empfohlen, wobei über die Häufigkeit von Pilzpneumonien unterschiedliche Daten vorliegen und Pilzpneumonien in unserem Obduktionsgut selten sind [40]. Während eine präexistente systemische Therapie mit Kortikosteroiden aufgrund der immunsuppressiven Wirkung einen Risikofaktor für schwere COVID-Verläufe darstellt, senkt eine Therapie mit Dexamethason die Mortalität über die Unterdrückung der überschießenden Entzündungsantwort [45]. Anti-Zytokin-Strategien gegen IL‑6 und TNF-alpha haben bisher keinen durchschlagenden Erfolg gezeigt [34]. Antivirale Therapieansätze, im speziellen der Einsatz von Remdesivir, Hydroxychloroquin, Lopinavir und Interferon beta-1a hatten trotz anfänglichem Optimismus keinen signifikanten Einfluss auf die Mortalität [46]. Der Einsatz von Rekonvaleszentenplasma hatte bei schweren Verläufen bisher keinen klaren Erfolg [37], während der Einsatz monoklonaler Antikörper gegen SARS-CoV‑2 in der Frühphase der Infektion derzeit noch untersucht wird. Die größten Hoffnungen ruhen derzeit zweifellos auf der Wirksamkeit der entwickelten Impfstoffe gegen SARS-CoV‑2 und der raschen Durchimpfung eines Großteils der Bevölkerung [16].

## Fazit für die Praxis

- COVID-19 führt vor allem im Bereich der Lungen zu schweren Organveränderungen in Form eines diffusen Alveolarschadens mit Übergang in eine organisierende Pneumonie.
- Wesentliche häufige letale Komplikationen sind Thrombosen kleiner und mittelgroßer Pulmonalarterien. Daraus resultieren Lungeninfarkte und bakterielle Bronchopneumonien.
- Pulmonale Thromboembolien sind eine weitere wesentliche Komplikation. Sie finden sich auch ohne klinisch manifeste tiefe Beinvenenthrombosen.
- Die gastrointestinale Manifestation von COVID-19 scheint insbesondere mit einer ischämischen Darmschädigung einherzugehen. Diese könnte sowohl durch Schock als auch durch lokalisierte Thrombosen entstehen.
- Bei letalem Verlauf ist fast immer eine schockbedingte Schädigung des Tubulusapparats der Niere vorhanden.
- Erkenntnisse aus Obduktionsstudien haben insbesondere die Prophylaxe von Thrombosen und Pneumonien beeinflusst.
